# Shall the Profession Do Contract Work Cheaper Than for the Public?*Read before the Brazos Valley Medical Society, May, 1906.

**Published:** 1906-10

**Authors:** J. P. Oliver

**Affiliations:** Caldwell, Texas


For Texas Medical Journal.

### Shall the Profession do Contract Work Cheaper Than for the Public?*

J. P. OLIVER, M. D., CALDWELL, TEXAS.

I shall discuss this subject under two heads:

*First.* The civic relation of the profession to the State and public generally and the social and legal relations to their patients.

*Second.* The power of corporate influence over medical men in their employ, and the meager compensation paid them for their services generally.

Perhaps there is no subject before the professional mind of today of greater magnitude in all of its bearings than the one under consideration; from the viewpoint of one who has nearly reached the time allotted him on this earth by Holy Writ it would seem to be opportune for discussion.

As a civilian no man in my estimation equals the physician or surgeon in civic righteousness; there are exceptions to this, we all know, but in their aggregate, I may be too optimistic in my views and place the civic estimate of medical men too high; but as my experience embraces about half a century along this line in the medical profession, it should be entitled, to say the least, to some consideration by my associates in the profession.

When this country calls to arms her chivalrous sons to whip a threatening or invading foe he quickly dons the surgeon's sash and bares his breast to the missiles of the enemy and various vicissitudes of camp life, and the diseases incidental thereto; when epidemics of every character are decimating by hundreds or thousands the population of the country and cities, who is the first to engage the enemy from every point until the battle is won, but oftentimes at the cost of the lives of many valuable medical men; in supporting the government under which they live, financially. I do not believe any man or set of men contribute more cheerfully of their means than does the medical profession.

On the other hand, when the State or county wishes their services, especially the county, to work for paupers and criminals, contract work is offered to the lowest bidder and in my county is generally given to the man who bids $1 a visit; sometimes this work is given to men who have never seen the inside of a medical

*Read before the Brazos Valley Medical Society, May, 1906.

college, much less having studied there to qualify himself for medical work. My point is to try to get the medical profession generally not to do contract work for the State, county nor corporations for less compensation than for their private patients.

It is not extravagant to say that the medical profession, of which I am a member, does more charitable work than any other profession, perhaps all others combined, I care not of what calling.

When the State calls the medical man to testify in the courts it would seem that the State forgets (or does not care) that the medical man's stock in trade is his medical or surgical knowledge, and as corporate companies, embracing common carriers, such as railroads and other commercial enterprises, are contending vigorously by any means at their command before the courts and legislative bodies for a reasonable precentage on their investments, the physican is paid a mere pittance for his services before the courts of whatsoever resort that he is required to give testimony in. This should not be the case. The Legislature should make an emergency appropriation to be used by proper authority in such cases, and reasonable compensation rendered to the physician or surgeon whose services have been rendered to the State or county. The high estimate which I have placed upon the members of the medical profession suggests at least what should be the relation to their patients, socially, morally and legally; no man or woman, it matters not what their position in society may be or has been, are so closely related to the sanctity of the family altar as the physician or surgeon; to him are confided the profoundest secrets of the family altar, from the bridal chamber to the closing deathbed scene; hence, it is in order to set some ideal to which members of our profession should attain.

First I would suggest that medical men, from an ideal point of view, should not only be civically righteous, but spiritually righteous; also both civic and Christian gentlemen. But as there are many members of the profession who are civic gentlemen, but not Christians, many of whom stand at the head of their profession, no criticism is intended but suggestive of the highest ideal medical man. If every member of the medical profession were such ideal men as suggested above, we should have no criminal abortion cases, no womb veils, no tubes with buttons to close the os, and the Anglo-Saxon race, instead of being in a decadent condition, would rise in its birth rate and not leave the race problem to the Latin-Teutonic and colored races.

The very sanctity of the family circle suggests the probity vested in the medical attendant, and the man who would betray any of the professional secrets is beneath the dignity of the medical profession. Even the law protects the physician and patient in this respect. But, alas, how many beautiful women have been sent to untimely graves by the professional abortionist, and the vast amount of gynecological work being done on account of prevention of conception.

There has never been an hour in the history of this government, and perhaps no other government, when corporate organizations exercised so much power as they do today. They virtually dictate, through the heads of their different bureaus, the major part of the policies of the government, both home and foreign, especially the financial part. Never before in the world's history have men become so fabulously rich, by controlling the various resources of the country. The masses are almost completely at the mercies and bidding of corporate influence in some form or another. Their organizations are so complete that from their home offices they can and do dictate any policy to their interest, and they have brought many medical men under their control by contract services; some perhaps are well paid, but in nowise like presidents, secretaries, and chief actuaries, yet, somehow, we medical men, and the legal profession also, love to work for them, some at minimum stipulated salaries or compensation. If any man is skeptical along this line let him review the investigations of the various insurance companies in New York and see how the policyholder's money has been used and abused, and then boast that Congress does not have the power to control them; their presidents drawing $150,000 annually; secretary $85,000 to $90,000, actuaries in proportion, besides their contributions for political purposes.

The companies' medical examiners in Texas and elsewhere are paid the pitiful sum of from $3 to $5 for their examinations of applicants for one to ten thousand dollar policies. Some of those companies have sent out bulletins cutting the $5 examinations to $3.

"In the corruption and distorted greed revealed in the insurance exposures of the year it is worthy of note that the medical officers and examiners of the companies have come out of the investigation with clean hands and untarnished reputation; it would thus appear that in the complicated organization of the large insurance companies their medical departments at least have been honestly and efficiently conducted, for them there is no word or censure or blame in the report of the Armstrong committee to the New

York Legislature. The change which has been made in appointing medical examiners in New York, and perhaps elsewhere, has in many instances been to the prejudice of the physicians, and is also, we believe, to the disadvantage of the real interest of the companies. Inexperienced and recent graduates in medicine, whose necessities are such that they can be obtained for the salary of a clerk, and are now appointed, and they are trained in the companies' offices to accept conditions which are degrading and which make it practicably impossible for the young physician ever to obtain practice. He is often obliged to remain for long hours at the down town offices of the company or is sent about the city at the behest of the agents to keep appointments which have been made for him and as to which he is not consulted. The young insurance doctor, unless he is a man of unusual stamina, becomes gradually more and more dependent upon the meager salary which he has fondly imagined to be only a help in tiding over the difficult period of waiting for patients; he drifts into middle age, still clinging to his monthly and stipulated salary; and at a time in life when he has lost initative and courage to make a change he discovers that it is too late, that he has given up an honorable and independent profession to become the mere tool of a corporation."—*New York Journal.*

But thanks be to the giver of all good, the reaction among the medical examiners all over the United States, both State and interstate, has begun. The profession seems to be aroused as never before to the relation they sustain to corporate organizations, and from Maine to the golden peaks of California, from the snow-capped mountains of Colorado to the gentle breezes of the Gulf, the universal cry is given every medical man not to examine for insurance companies for less than $5; and, my brethren of the Brazos Valley Medical Association, let us stand pat and do not work for any corporation for less compensation than we do for our private patients, and I will request that we do no contract work for either State or county for less money than we do for our private patients. Some of the corporate organizations under contract work require that we make the usual charges in any case, whether surgical or not, and then deduct one-half. I for one do not want any free pass if I ride on the railroad. I shall pay for it at their own prices. If I work for these companies I demand reasonable compensation, not less than I obtain from my private patients. By this I am willing to live and die.

The Texas State Medical Association has spoken through its

House of Delegates at the Fort Worth Convention in no uncertain tones; so has Potter county and many other county societies. This is why I thought it timely to raise my voice, though it be feeble and may not be observed, to warn the young men of the medical profession against corporate greed and their corrupting influence in this great commonwealth. It is my opinion that they are as dangerous to the body politic, to the moral and social fabric of this government, to professional men, especially of law and medicine, as the assassin's bullet in the midnight hour.

In closing my remarks, I wish to call the attention of the younger men of the profession to some points which may be profitable in future life. The older men of the profession, like Drs. Collard, Parker and myself and others, will soon have passed off of the stage of action. To you we wish to bequeath one of the grandest, most honorable and independent professions in the world's history; a profession fraught with many possiblities to the younger men of scientific and practical medical and surgical knowledge. Within the two last decades the evolution of the medical and surgical sciences has been wonderfully phenomenal. In our college days we were not taught asepsis and sepsis, biology, insectology, micro-organisms, serums, nor laboratory work of any kind, not even the tertian quid of the stegomyia fasciata, and human organism nor do we profess to know but very little about them yet personally. But with the advanced thought of the age we commend this honorable profession to your careful study and evolution.

We know that the young men, especially those of Texas, are the pride and future hope of this great empire State; and as we from natural causes will soon take our departure from among you, we request that when you have served this honorable profession as long as we have and as faithfully as we, that your efforts will have solved many doubtful problems now at issue and that you will bequeath it to posterity fraught with as many possibilities as now; and our prayer is that your efforts will ever be onward and upward, remembering that there is always room on the topmost rounds of fame in the medical and surgical sciences. Last, but not least, let your civic righteousness be as advised by the Apostle Paul to the Gentiles: "Not only avoid evil, but the very appearances of evil."

